# Biodiversity breakpoints along stress gradients in estuaries and associated shifts in ecosystem interactions

**DOI:** 10.1038/s41598-019-54192-0

**Published:** 2019-11-26

**Authors:** Emily J. Douglas, Andrew M. Lohrer, Conrad A. Pilditch

**Affiliations:** 1 George Mason Centre for the Natural Environment, The University of Auckland, Private Bag 92019, Auckland, 1142 New Zealand; 2 National Institute of Water and Atmospheric Research, PO Box 11-115, Hillcrest, Hamilton 3251 New Zealand; 3 School of Science, University of Waikato, Private Bag 3105, Hamilton, 3216 New Zealand

**Keywords:** Element cycles, Biodiversity, Ecosystem ecology, Ecological networks, Marine biology

## Abstract

Denitrification in coastal sediments can provide resilience to eutrophication in estuarine ecosystems, but this key ecosystem function is impacted directly and indirectly by increasing stressors. The erosion and loading of fine sediments from land, resulting in sedimentation and elevated sediment muddiness, presents a significant threat to coastal ecosystems worldwide. Impacts on biodiversity with increasing sediment mud content are relatively well understood, but corresponding impacts on denitrification are uncharacterised. Soft sediment ecosystems have a network of interrelated biotic and abiotic ecosystem components that contribute to microbial nitrogen cycling, but these components (especially biodiversity measures) and their relationships with ecosystem functions are sensitive to stress. With a large dataset spanning broad environmental gradients this study uses interaction network analysis to present a mechanistic view of the ecological interactions that contribute to microbial nitrogen cycling, showing significant changes above and below a stressor (mud) threshold. Our models demonstrate that positive biodiversity effects become more critical with a higher level of sedimentation stress, and show that effective ecosystem management for resilience requires different action under different scenarios.

## Introduction

Excessive nutrient and sediment inputs from land are threatening the wellbeing of marine ecosystems worldwide^1–4^. Coastal soft sediment ecosystems, especially estuaries, are areas of considerable transformation of energy and matter, and are hotspots for biogeochemical processes between the land and the sea. Understanding estuarine and coastal marine nitrogen cycling and nitrogen removal pathways is particularly important to coastal environmental managers due to the prevalence of eutrophication symptoms. Both allochthonous and autochthonous organic matter and nutrients support the productivity of estuarine and marine food webs, regulated by estuarine soft sediment ecosystem ‘reactors’ that process and recycle a significant amount of this material. These sediment ‘reactors’ mineralise organic matter into ammonium which can then be released to the water column fuelling phytoplankton and macroalgal growth, or assimilated by microphytobenthos fuelling benthic primary production (which creates new autochthonous organic matter) or, transformed in the sediment through recycling and removal pathways such as coupled nitrification-denitrification, anaerobic ammonium oxidation (anammox), and dissimilatory nitrate reduction to ammonium (DNRA). Nitrogen cycling in marine sediments is complex, dynamic in space and time, and dependent on multiple interrelated ecosystem components. Improved understanding of the relationships and interactions between ecosystem components (e.g. biodiversity measures) and key ecosystem functions (e.g. denitrification) in these soft sediment reactors is required for effective management for ecosystem resilience.

Denitrification, the removal of bioavailable nitrogen, is a key function of coastal soft sediment habitats that can provide ecosystem resilience to excess nutrient enrichment. Estuary denitrification is dependent on local environmental variables (e.g. ammonium, nitrate and oxygen concentrations, temperature, salinity, sediment size, and sediment organic matter content) and macrofaunal community composition^5^, and it is sensitive to stressors^6^. However, because most studies are restricted in space and time, and denitrification measurements are logistically difficult as well as costly, few studies measure denitrification across environmental gradients and include ecosystem variables such as sediment composition or macrofaunal community and biodiversity (for exceptions see^5,7,8^). Despite a substantial body of literature on the drivers of aquatic denitrification, much of the research that underpins our current mechanistic understanding is based on laboratory or mesocosm studies, and there is a lack of empirical field studies^9,10^. For example, knowledge of the influence of sediment type on denitrification rates comes from flume or core experiments and models (e.g.^11–13^), and the role of macrofauna for denitrification has been primarily demonstrated using core incubations with manipulated faunal compositions e.g.^14–16^. As a result, it is difficult to generalise and scale up studies of denitrification. Furthermore, the interrelationships among known controls of denitrification (i.e. sedimentary environment and biodiversity measures) are not well understood, nor do we know about the potential indirect effects on denitrification, the effect that changes in these relationships could have on overall ecosystem resilience, or how this network of interactions may change when ecosystem stressors increase or interact (e.g. multiple stressor effects caused by increasing sedimentation and biodiversity loss).

Bioturbation by resident macrofauna facilitates many biogeochemical processes in marine sediments^17^ meaning that macrofaunal community composition and biodiversity are fundamental to sediment nitrogen cycling, but these important sediment-macrofauna relationships are often context dependent and vary with sediment type^5,18,19^. Similarly, microphytobenthos can have a role in regulating denitrification and other sediment nitrogen transformations through oxygenating surface sediments and changing redox gradients, and it too is influenced by the sedimentary environment^20,21^. Sediment mud content is a key stressor in coastal ecosystems and is increasing because of land use intensification and climate change^22–24^. Studies have demonstrated that increasing sediment mud content affects biodiversity, macrofaunal community composition, macrofaunal behaviour, microphytobenthic biomass, and ecosystem functions (including those associated with nitrogen processing), and that these changes can be abrupt, occurring at certain ‘breakpoints’ or levels of mud content^18,25–28^. We therefore expect that increases in sediment mud content will alter relationships between benthic biodiversity and ecosystem functions such as microbial nitrogen processing^29^.

Environmental change and increasing stressors requires resilience based management of ecosystems^30,31^, but this cannot be done without understanding ecosystem components, their interactions, and how they contribute to processes like denitrification that provide resilience. Structural equation models (SEMs) use path analysis to evaluate networks of multiple known or theoretical causal relationships to understand complex systems^32,33^. SEMs can be used to model interaction networks and predict and understand interactions between organisms, multiple environmental variables, and ecosystem functioning e.g.^34–36^. These approaches have yet to be fully utilised to understand ecosystem functions and biogeochemical processes that may contribute to resilience^37–39^. Correlative approaches that use real world ecological data to identify connections between environmental variables and ecosystem functions enhance the generality of ecological experiments^40^. Here we used SEMs to investigate the ecosystem components that contribute to microbial nitrogen processing (measured as denitrification enzyme activity (DEA), see^41,42^), and the role of biodiversity in interaction networks. The DEA assay is conducted *in vitro* under optimal conditions for denitrification and uses acetylene to block the N_2_O to N_2_ step in denitrification so that N_2_O production can be quantified (easier than measuring fluxes of atmospherically abundant N_2_). The assay conditions are not representative of real world conditions, so the obtained results provide an enzymatic proxy for the denitrifying bacterial community of a sediment sample, rather than a direct measure of denitrification rate. Because denitrifying bacteria can persist in the sediment for several months or years^43,44^ and quickly activate in response to favourable conditions^45^, DEA provides a useful integrative measure of the denitrification history of the sediment.

Using data from five different estuaries spanning gradients in biotic and abiotic variables, we analysed for thresholds in sediment mud content and assessed differences in interaction network architecture between sandy and muddy ecosystems using SEMs to evaluate changes in ecosystem dynamics and effects on microbial nitrogen processing (DEA). Specifically, we wanted to determine whether (and how) breakpoints in biodiversity with increasing stressors corresponded to changes in DEA. We predicted that there would be thresholds in biodiversity measures with increasing sediment mud content, and that ecosystem interaction networks associated with DEA would differ in sandy versus muddy sediments, due to losses or changes in important network linkages associated with increasing sedimentation stress. This study uses a large dataset encompassing broad spatial extent with a novel analysis approach that contributes significantly to our understanding of estuarine denitrification and highlights the importance of context specific interactions. By investigating stressor thresholds and characterising interaction network changes across thresholds, this study provides a step towards generalising and scaling up ecosystem functions and measures of ecosystem services, in the context of increasing stressors and potential tipping points^46^. Additionally, our approach enables visualisation of how ecosystem response to changes, such as biodiversity loss, may manifest under different stressor scenarios.

## Results

**Figure 1:**
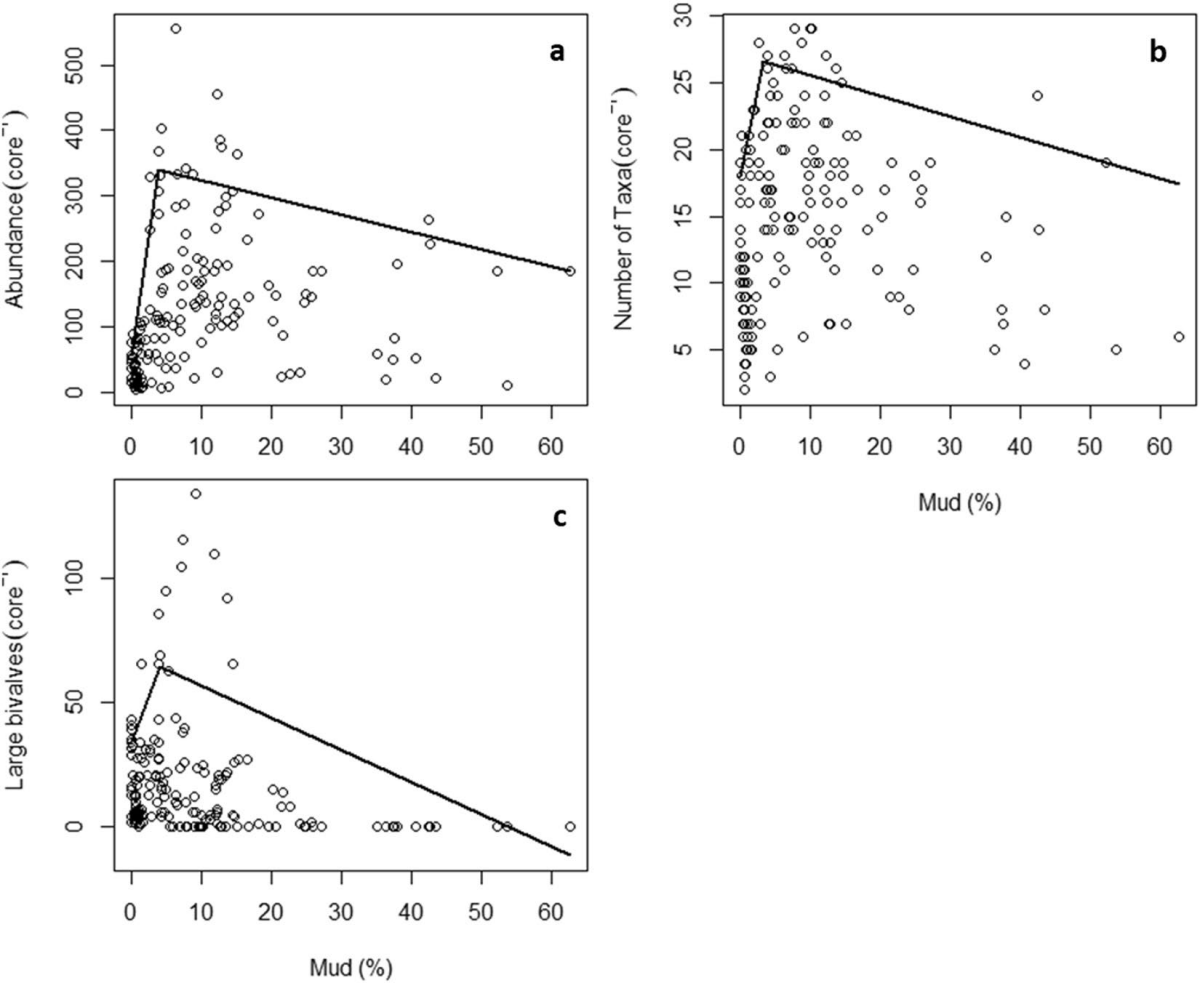
Bivariate scatterplots of biodiversity measures showing breakpoints in relationships with sediment mud content from 171 data points across the five estuaries. Lines represent segmented quantile (τ = 0.9) regressions with breakpoint estimates and standard errors for (**a**) macrofaunal abundance; 3.79 ± 1.02, (**b**) number of taxa; 3.21 ± 1.58, and (**c**) number of large bivalves; 4.02 ± 3.61, with overall mean of 3.6%.

**Figure 2:**
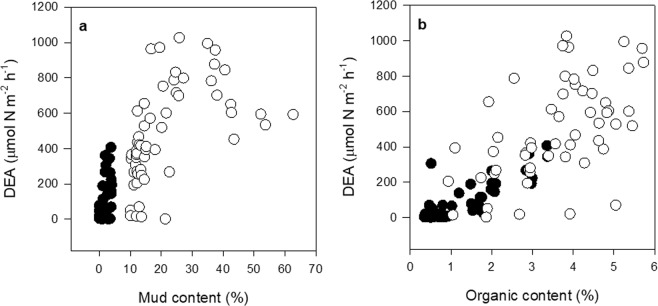
Bivariate scatterplots showing relationship between DEA and (**a**) mud content and (**b**) organic content. The sandy sediment (<4% mud, n = 75) data are indicated by (●) and the muddy (>10% mud, n = 57) data by (○).

**Figure 3:**
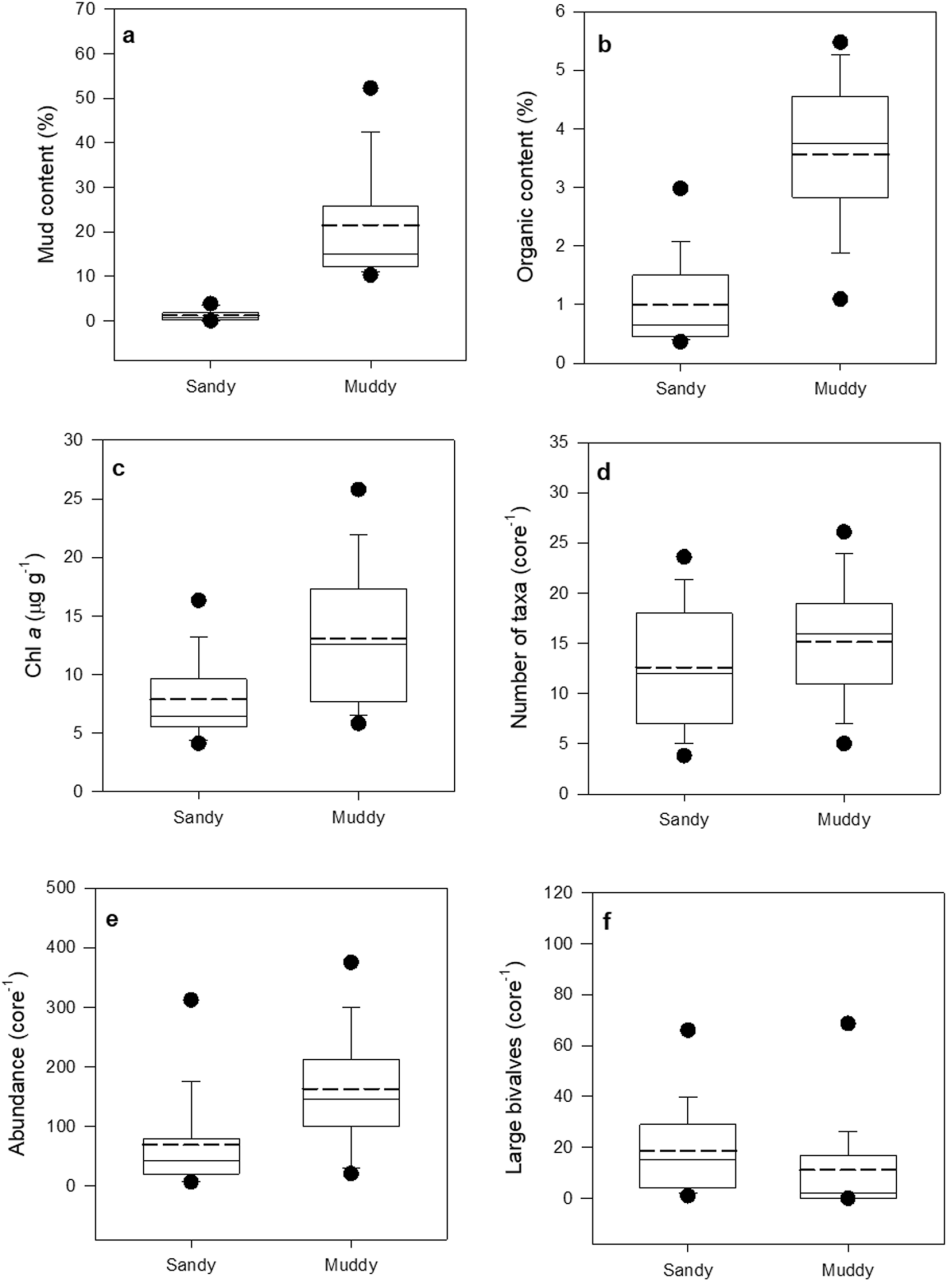
Box plots for ecosystem components (**a**) mud content, (**b**) organic content, (**c**) Chl *a*, (**d**) number of taxa, (**e**) macrofaunal abundance, and (**f**) number of large bivalves in the sandy (<4% mud, n = 75) and muddy (>10% mud, n = 57) datasets. Boxes show 25^th^ and 75^th^ percentiles, whiskers the 10^th^ and 90^th^ percentiles and black dots the 5^th^ and 95^th^ percentiles. Solid line is median and dashed line is mean.

Quantile regressions (90%) of biodiversity measures and sediment mud content showed positive relationships at very low levels of mud (~4% on average), after which the relationships became negative (Fig. 1). DEA increased with both mud and organic content, and although mud and organic content are strongly correlated (Pearson’s R = 0.69, p < 0.001), the sandy (<4% mud) and muddy (>10% mud) data subsets showed overlapping values of organic content as well as other model response variables (Figs. 2 and 3).

**Figure 4:**
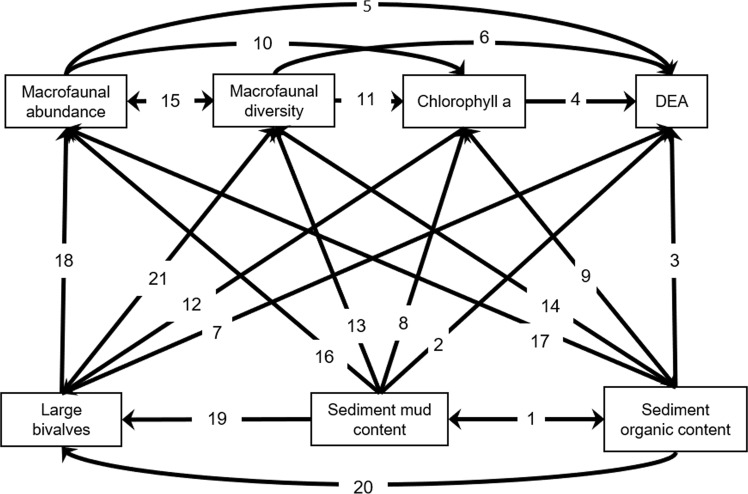
Conceptual basis model defining possible relationships between measured variables. Path numbers correspond to known relationships described in Supplementary Table S2. Unidirectional arrows represent a causative relationship whereas bidirectional arrows represent correlation.

**Figure 5:**
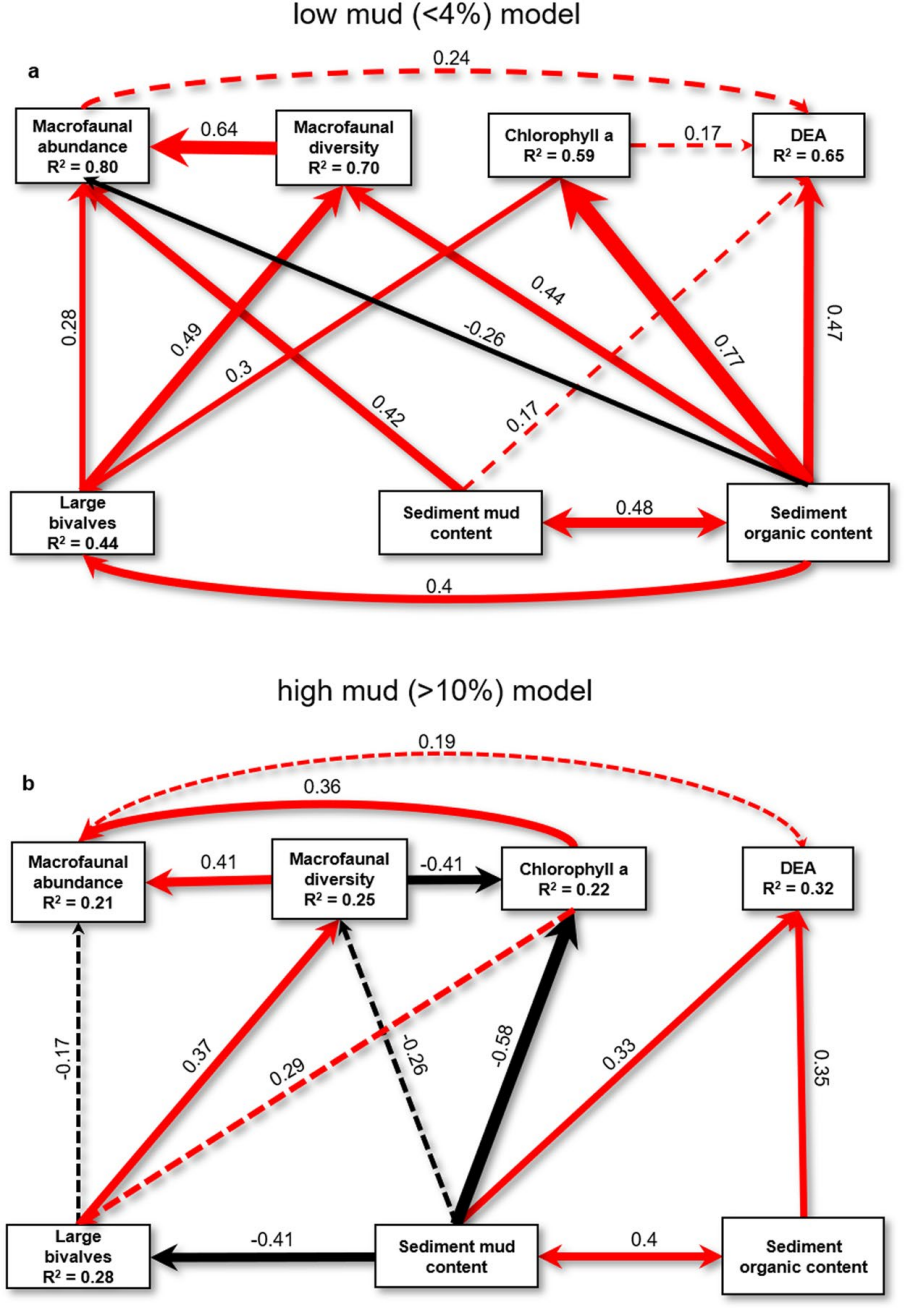
Structural equation models depicting ecosystem interactions contributing to DEA in (**a**) sandy (<4% mud) and (**b**) muddy (>10% mud) sediments. R^2^ values are shown for each response variable and path thickness is scaled with the path coefficient. Red paths indicate positive effects whereas black paths indicate negative effects. Path coefficients are significant at p < 0.05 except where lines are dashed, these non-significant paths were retained for overall model stability. Double headed arrows indicate factor covariance. Sandy model test statistic χ^2^ = 4.52, p = 0.72, n = 75, df = 7, muddy model test statistic χ^2^ = 3.36, p = 0.91, n = 57, df = 8.

The conceptual basis model used for our structured equation model analyses demonstrates the high potential for multiple direct and indirect controls on DEA, and the highly connected system that contributes to variability in DEA (Fig. 4). Interaction networks were significantly different in sandy compared with muddy systems (Fig. 5), and all response variable R^2^ values decreased in the muddy model (Fig. 5). Most notable was the reduction in significant positive network linkages (from 9 to 6) and increase in the number of significant negative network linkages (from 1 to 3) from the sandy to muddy model (Fig. 5). Sediment organic content changed from having a strong direct effect on DEA in the sandy model, as well as being a potentially integral driver of many ecosystem components with indirect effects on DEA, to having only one direct (and weaker) effect on DEA in the muddy model. Compared with the sandy model, in the muddy model there were more significant direct effects and more indirect effects on DEA.

Models showed that in the muddier sites, mud had significant negative effects (both direct and indirect) on biodiversity measures that were absent in the sandy sites. The positive influence of macrofaunal abundance appeared to be slightly more important for DEA in sandier (non-significant direct effect) as opposed to muddier sediments (weaker non-significant direct effect), indicating a reduced coupling of DEA to macrofauna (Fig. 5). Although mud had a strong positive direct effect on DEA in the muddy model, there were at least 4 pathways where negative effects of mud on ecosystem components (especially macrofauna) had indirect negative effects on DEA (Fig. 5b).

## Discussion

Our dataset was consistent with other studies demonstrating changes or breakpoints in biodiversity measures with increasing mud content^18,26,29,38^. The dataset showed a high degree of variability as expected in real-world ecological systems^47–49^; for example, macrofauna-sediment relationships showed factor-ceiling-like relationships (Fig. 1). This high degree of variability can make assessing or detecting changes in important ecological relationships and interactions difficult, however SEMs and interaction networks help us disentangle the relationships present among this variability and understand the underlying ecosystem processes^50^. The differences in ecological interaction networks observed for <4% and >10% mud content demonstrated how potentially important ecological relationships can change with the muddying of intertidal estuarine habitats. Our results are consistent with other work showing ecosystem changes associated with increases in sediment mud content including; a threshold response at 20% mud^28^, reductions in macrofaunal traits based indices above 10% mud^51^, and functional shifts in the relationship between a key consumer and the abundance of microphytobenthos above and below 15% mud content^49^.

Although muddy sediments are typically organic rich, sediment mud content may affect ecosystem functioning, especially denitrification, in a concentration-dependent or threshold type manner due to interactions between mud and OM and their combined effects on macrofauna. Strong correlations among these drivers of DEA (i.e. mud and organic content) make it hard to decipher whether effects of changing sedimentary environment are due to physical effects caused by higher proportions of finer sediments, or chemical effects such as supply of bioavailable nitrogen and carbon from breakdown of organic matter. Muddy sediments tend to have high organic content and therefore pore water nitrogen concentrations (from organic matter breakdown), but slower rates of pore water advection compared with sandy sediments^52^. For microbial nitrogen cycling this means that although there is higher supply of inorganic nitrogen in muddy sediments, rates of nitrogen transformation are limited by the movement of solutes throughout the sediment, whereas in sandy sediments there is faster pore water flow and solute transport, but rates of nitrogen processing are limited by lower solute concentrations^12^. Our models reflect this; the influence of mud versus organic content on DEA differed in our sandy (<4% mud) and muddy (>10% mud) datasets. In sandy systems, organic content is an important driver of DEA (and other ecosystem response variables), suggesting that DEA is more limited by the quantity of inorganic nitrogen substrates. Whereas our muddy sediment SEM suggests that DEA is more limited by the supply of resources to specific sediment horizons where they can be transformed (due to the slow speed of diffusion through the smaller pore spaces in cohesive muddy sediments). The influence of sediment organic content on DEA is less in the muddy model, and the effect of macrofaunal abundance on DEA remains an important linkage, despite the lower path coefficient and much lower explained variance of macrofaunal abundance as a response variable (R^2^ = 0.21). It follows that ecosystem components that enhance sediment mixing (i.e. bioturbation), such as macrofaunal abundance, may become relatively more important in muddy sediments because the bioadvection of solutes facilitates the supply of resources to DEA (and other biogeochemical processes).

Bioturbation is an important ecosystem function carried out by benthic macrofauna that is known to significantly enhance microbial nitrogen processing^17^. The effect of bioturbation on biogeochemical fluxes is enhanced by the abundance of key species (large taxa, such as *A. stutchburyi* and *M. liliana* in the present study, that mix and mobilise sediments via their movements and feeding behaviours), community abundance, species richness, and functional diversity^5,53–56^. These models suggest that the link between macrofaunal abundance and DEA represents a critical positive relationship (and link between other important influences on DEA including macrofaunal diversity and microphytobenthic biomass (Chl *a*)), and that the strength of this link is reduced with increasing stress (mud content) and the associated changes in the overall ecosystem interaction network.

These network models allow us to characterise how ecosystems respond to increasing stressors at a system level by helping to identify the mechanisms behind ecosystem function response to perturbation, as well as understand the consequences of how further anthropogenic pressure may alter the system, its interactions and ecosystem functions^57^. Our interaction network analyses show how changes in an ecosystem, such as shifts in macrofaunal community, may have effects on other parts of an ecosystem, or ecosystem functions, and that shifts may have differing effects under different scenarios or stress regimes. For example, overharvesting of shellfish (large bivalves) may have several indirect effects on microbial nitrogen cycling (DEA), but these effects are likely to be more severe in ecosystems with elevated mud content. Similarly, loss of biodiversity and abundance of macrofauna, will have differing effects on ecosystem dynamics, and therefore microbial nitrogen processing, in estuaries that have high sedimentation levels (high mud content) than those that are sandier. This result may be indicative of how important ecological relationships may change with increases in other stressors, however further analysis including measures of other stressors (e.g. nutrient enrichment) and ecosystem functions (e.g. primary productivity) is required to test this. Comparing the two models reveals that higher mud content resulted in a reduction in the amount of explained variability in ecosystem components (including DEA), and this can be symptom of increased ecosystem stress^58–61^. Increased variability in ecosystems can also be indicative of an approaching tipping point or regime shift^62–64^, thus our approach could be used as an early warning indicator as well as a tool for mitigating such changes.

It should be noted that many healthy estuaries are likely to fall within the 4–10% mud content range not analysed in our study, and <10% mud content would ordinarily not be considered impacted by sedimentation. We do not have a sufficient number of data points within the 4–10% mud content range to build a SEM, but we would anticipate some differences in linkages compared with the low mud model. Sediment stability (cohesive to non-cohesive), biogeochemical processes and macrofaunal communities and their inter-relationships change substantially across this range of mud content^25,29,65^ and we would expect the interaction network to reflect this accordingly. With the data we do have between 4–10% mud content Pearson’s correlation coefficients revealed strong positive correlations between large bivalves and DEA, and Chlorophyll *a* and DEA, suggesting that biological and macrofaunal ecosystem components are again key drivers of functionality. Without a larger dataset we cannot speculate on whether key functional linkages would be lost within this range but it is worthy of further study.

Investigating ecosystem resilience and thresholds is increasingly important in an era of unprecedented anthropogenic change. Detecting and responding to major ecosystem changes before they occur is a critical challenge for ecologists and managers of coastal ecosystems^64,66,67^. However, the way to quantitatively assess resilience and detect thresholds (from a management perspective) is less clear and requires more research^68^. Our investigation has demonstrated that at different levels of a stressor (sediment mud content) the architecture of ecosystem interaction networks changes with consequences for ecosystem function. Our structural equation model analyses support previous experimental work demonstrating the interdependence of estuary microbial nitrogen processing on both physical (sedimentary environment) and biological (macrofaunal community and biodiversity) ecosystem variables^5,12,13^. Furthermore, we show evidence for loss of resilience of microbial nitrogen cycling with increasing sedimentation stress through changes in interaction networks, increased negative effects on potentially important network linkages (especially biodiversity related), and increased dependence on these positive biodiversity effects. Our models showed that the mechanisms of ecosystem components acting on DEA are different under different levels of sedimentation stress, and therefore managing for ecosystem resilience to nutrient enrichment requires different action in different scenarios.

This study confirms that there is high interdependence of microbial nitrogen processing on other ecosystem components and their interactions, meaning that resilience to nutrient enrichment will be highly responsive to ecosystem changes such as increasing sediment mud content. Interaction networks reveal that DEA is more tightly linked, or dependent on, the macrofaunal community in muddy sediments than in sandy sediments. In agreeance with many other studies, this means that the effects of ecological relationships, especially those involving biodiversity components, are context dependent, and more imperative to ecosystem resilience when levels of a stressor are higher^69–71^. This research is an example of using data from site-specific studies to scale up and increase the generality and broadness of understanding of how ecological relationships and interaction networks operate over greater environmental gradients and changes in ecosystem components. Preservation of benthic macrofaunal community biodiversity is essential to mitigate the effects of increasing stressors on nitrogen cycling and ecosystem resilience, and this has greater importance in ecosystems already under sedimentation stress. Understanding these general patterns and how they change across environmental gradients, and/or with increasing stressors provides a way forward for management and conservation when working with ecological datasets that are inherently heterogeneous.

## Methods

**Figure 6:**
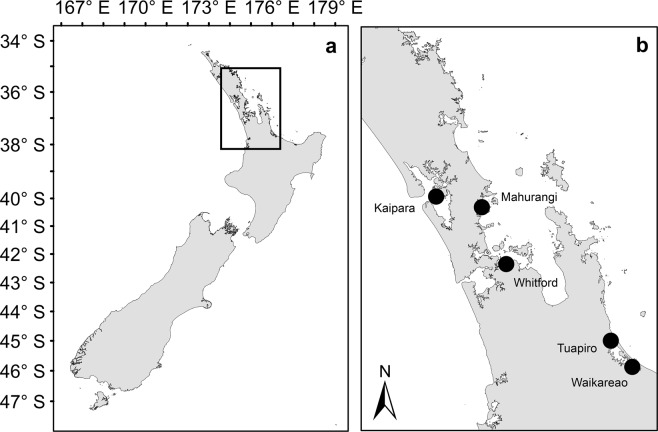
Map of (**a**) New Zealand and (**b**) locations of the five estuaries from which samples were taken.

Data were compiled from two published (the un-manipulated/ambient plots in Douglas, *et al*.^69,72^) and three unpublished surveys^73,74^ carried out in five northern New Zealand estuaries with extensive intertidal areas, and catchments with variable land use (Fig. 6, Supplementary Table S1). In total, the dataset consisted of samples collected from 171 plots (1 m^2^) during austral summer months (December-March) between 2013 and 2018. The estuaries contained overlapping gradients in abiotic (especially sediment properties) and biotic ecosystem components allowing data to be pooled and analysed for patterns across estuaries.

### Denitrification enzyme activity—the primary response variable of interest

Methods for Denitrification Enzyme Activity (DEA) sample collection and assays are described in full in Douglas, *et al*.^69^, but briefly, 5 replicate sediment cores (0–5 cm depth, 5.3 cm dia.) were taken randomly from each plot and pooled. Unfiltered seawater was collected from each site, and water and sediment were stored on ice for transport to the laboratory where they were stored at 4 °C. Assays were conducted at room temperature (20 °C) within 48 h of sample collection using the chloramphenicol amended acetylene inhibition technique^41,75^. Assay slurries contained 30 mg L^−1^ C as glucose and 10 mg L^−1^ N as KNO_3_. Headspace gas samples (6 mL) were collected from each assay over a 2 h time course (10, 30, 60, 120 min) under anoxic conditions with constant mixing (125 rpm) after the addition of acetylene (which blocks conversion of N_2_O to N_2_). Gas samples were analysed for N_2_O concentration using a Varian CP 3800 gas chromatograph equipped with a HayeSep D column and an electron capture detector (ECD). DEA rates (i.e. N_2_O production) were expressed per unit area of sand flat (µmol N m^−2^ h^−1^ ^69^).

### Ecosystem properties

Paired with each DEA measurement, sediment properties and microphytobenthic biomass were characterised from 5 pooled surface sediment samples (2.6 cm dia., 0–2 cm depth), and macrofaunal invertebrates >500 µm longest dimension were sampled using 13 cm dia., 15 cm depth sediment cores (n = 2 per plot in Kaipara Harbour, n = 1 per plot at all other sites)^76^. For measurements of sediment mud content (Mud), organic matter was first removed from samples using 10% hydrogen peroxide, then samples were analysed using a Malvern Mastersizer 2000^77^ to derive the proportion of mud (particle sizes <63 µm). Sediment samples were dried to a constant mass (60 °C, 48 h), then organic content (OC) was determined by weight loss on ignition (550 °C for 4 h)^78^. Microphytobenthic biomass (Chlorophyll *a* (Chl*a*)) was determined after extraction of pigments from freeze-dried sediments using 90% acetone, then a Turner Designs 10-AU flourometer was used to measure fluorescence^79^. Preserved (50% isopropyl alcohol) macrofauna were stained with Rose Bengal and in the laboratory, all organisms were counted and identified to the lowest possible taxonomic level (usually species). Macrofaunal community variables (biodiversity measures) included total abundance (N), number of taxa (S), and number of the large bivalve species *Macomona liliana* and *Austrovenus stutchburyi* (LB; considered separately because of their substantial influence on ecosystem function on New Zealand sandflats^26,29,80^).

### Low versus high mud datasets

Our key question was how ecological interaction networks change with increased sediment muddiness and how this may affect DEA. This required the development of SEMs for sandy (low mud) and muddy situations. We created two datasets (sandy and muddy), given the following constraints. SEMs require a large number of data points, with the minimum number of data points determined by the number of nodes present in an initial conceptual basis model (see below). Any split of the data into sandy vs muddy required at least 55 data points in each subset. Further, we endeavoured to create two data sets with an approximately equal number of data points in each, as this was the most objective way to compare the significance of paths in sandy and muddy SEMs.

Bivariate plots of biodiversity measures with sediment mud content as the predictor variable were examined to investigate thresholds in mud content that could be indicative of changes in ecosystem interaction networks (Fig. 1). This was done using quantile regression and breakpoint analyses using packages ‘quantreg’^81^ and ‘segmented’^82,83^ in R 3.5.1^84^. Breakpoints in sediment mud content (the predictor variable) for each biodiversity measure (number of species, number of individuals, and number of large bivalves) were determined using breakpoint analyses of the 90^th^ percentile (τ = 0.9) quantile regression models (Fig. 2). The average breakpoint was determined to be 4% mud, which was then used to create a low mud content dataset (i.e., “sandy”, <4% mud, n = 75). A higher mud content dataset was created with values >10% mud content (i.e., “muddy”, n = 57). This value is based on previous work showing that DEA responds negatively to stress when there is >10% mud content (but not <10%)^72^, indicating potential changes in ecosystem interaction networks associated with DEA may occur around this point. Having disjunct datasets (i.e., <4% mud for sandy, and >10% mud for muddy) allowed us to present coherent results for mud levels above and below the 4–10% mud range, which literature suggests may encompass other ecologically significant thresholds^28,51,72,85^. Specifying a precise breakpoint position within this range was not required. Given our analytical goals and constraints (e.g., minimum dataset size, relatively balanced datasets), our approach was appropriate. Each dataset contained a minimum of four samples from each estuary, except for Mahurangi where there were only samples with >10% mud content. This minimised any confounding effects of ‘estuary’ on results.

### Conceptual basis for structural equation model

Structural equation models were built using the Lavaan package^86^ in R 3.5.1^84^ for the two datasets. Models were developed starting with a conceptual basis model (Fig. 4, Supplementary Table S2), and sequentially removing non-significant paths and checking modification indices (path changed required if modification index >5). Model fit was assessed using the χ^2^ statistic and considered acceptable when p > 0.05. The conceptual basis model for the SEM was defined according to known relationships between ecosystem components, and the influence of ecosystem variables on denitrification (Fig. 4, Supplementary Table S2). All variables were log (Mud, OC, Chl*a*, DEA) or square root (number of taxa, macrofaunal abundance, large bivalves) transformed prior to building SEMs.

## Supplementary information

Supplementary Information

## Data Availability

All data analysed during the current study is available in this published article and its supplementary information files.
