# Supplementary material for: Biodiversity breakpoints along stress gradients in estuaries and associated shifts in ecosystem interactions
Source: Sci Rep. 2019 Nov 26;9:17567. doi: 10.1038/s41598-019-54192-0 (PMC6879482; doi:10.1038/s41598-019-54192-0)
Supplement: Supplementary file 1 — Supplementary Information [file 41598_2019_54192_MOESM1_ESM.pdf]

Supplementary Information

Biodiversity breakpoints along stress gradients in estuaries and associated shifts in ecosystem interactions

Emily J. Douglas, Andrew M. Lohrer, Conrad A. Pilditch

Table S1 Site information and raw data.

Study	Site	Plot	Sample date	Location		Water depth	Temperature	Salinity	OC	Mud	Microphytobenthic biomass	Macrofaunal community			DEA
				Latitude	Longitude						Chl*a*	S	N	LB	
units				NZTM	NZTM	m	ºC	ppt	%	%	μg g^{-1}	n core^{-1}	n core^{-1}	n core^{-1}	μmol N m^{-2} h^{-1}
Waikareao	2	Q1	13/01/2013	5824126	1878123	Intertidal	20.6 ± 0.2	ND	2.9	10.4	10.7	19	141	3	366
Waikareao	2	Q2	13/01/2013	5824126	1878123	Intertidal	20.6 ± 0.2	ND	2.8	12.0	6.0	17	115	5	365
Waikareao	2	Q3	13/01/2013	5824126	1878123	Intertidal	20.6 ± 0.2	ND	2.9	11.2	12.6	14	100	3	266
Waikareao	2	Q4	13/01/2013	5824126	1878123	Intertidal	20.6 ± 0.2	ND	2.8	14.6	11.6	19	138	4	353
Waikareao	2	Q5	13/01/2013	5824126	1878123	Intertidal	20.6 ± 0.2	ND	2.9	11.1	10.5	19	102	5	194
Waikareao	3	Q1	13/01/2013	5824432	1877789	Intertidal	20.6 ± 0.2	ND	3.0	15.1	25.7	7	364	0	395
Waikareao	3	Q2	13/01/2013	5824432	1877789	Intertidal	20.6 ± 0.2	ND	2.9	13.3	25.7	11	299	0	281
Waikareao	3	Q3	13/01/2013	5824432	1877789	Intertidal	20.6 ± 0.2	ND	3.0	12.8	22.8	7	374	0	421
Waikareao	3	Q4	13/01/2013	5824432	1877789	Intertidal	20.6 ± 0.2	ND	2.9	12.2	26.4	12	457	1	270
Waikareao	3	Q5	13/01/2013	5824432	1877789	Intertidal	20.6 ± 0.2	ND	2.9	12.5	26.3	7	386	0	257
Waikareao	4	Q1	13/01/2013	5824939	1878310	Intertidal	20.6 ± 0.2	ND	3.5	12.3	17.1	22	296	19	612
Waikareao	4	Q2	13/01/2013	5824939	1878310	Intertidal	20.6 ± 0.2	ND	3.5	9.4	19.3	19	228	24	504
Waikareao	4	Q3	13/01/2013	5824939	1878310	Intertidal	20.6 ± 0.2	ND	3.7	16.4	17.8	21	260	27	571
Waikareao	4	Q4	13/01/2013	5824939	1878310	Intertidal	20.6 ± 0.2	ND	3.4	11.9	14.8	24	267	17	349
Waikareao	4	Q5	13/01/2013	5824939	1878310	Intertidal	20.6 ± 0.2	ND	3.6	13.4	21.7	19	306	21	416
Waikareao	5	Q1	13/01/2013	5824771	1878741	Intertidal	20.6 ± 0.2	ND	2.0	4.0	11.4	22	400	69	196
Waikareao	5	Q2	13/01/2013	5824771	1878741	Intertidal	20.6 ± 0.2	ND	2.0	3.8	13.6	26	411	43	159
Waikareao	5	Q3	13/01/2013	5824771	1878741	Intertidal	20.6 ± 0.2	ND	2.1	3.9	12.6	27	394	86	145
Waikareao	5	Q4	13/01/2013	5824771	1878741	Intertidal	20.6 ± 0.2	ND	1.9	5.2	13.8	24	252	63	216
Waikareao	5	Q5	13/01/2013	5824771	1878741	Intertidal	20.6 ± 0.2	ND	2.1	3.7	17.1	22	374	66	191
Tuapiro 1	1	Q1	11/02/2013	5846357	1859717	Intertidal	22.6 ± 0.2	ND	5.2	5.8	7.9	20	103	0	83
Tuapiro 1	1	Q2	11/02/2013	5846357	1859717	Intertidal	22.6 ± 0.2	ND	5.1	6.8	7.8	15	93	0	80
Tuapiro 1	1	Q3	11/02/2013	5846357	1859717	Intertidal	22.6 ± 0.2	ND	5.2	5.4	6.7	12	55	0	76
Tuapiro 1	1	Q4	11/02/2013	5846357	1859717	Intertidal	22.6 ± 0.2	ND	5.0	12.9	7.1	13	103	0	70
Tuapiro 1	1	Q5	11/02/2013	5846357	1859717	Intertidal	22.6 ± 0.2	ND	5.6	9.9	7.9	16	76	0	54

Study	Site	Plot	Sample date	Location		Water depth	Temperature	Salinity	OC	Mud	Microphytobenthic biomass	Macrofaunal community			DEA
				Latitude	Longitude						Chl*a*	S	N	LB	
units				NZTM	NZTM	m	ºC	ppt	%	%	µg g^{-1}	n $core^{-1}$	n $core^{-1}$	n $core^{-1}$	µmol N m^{-2} h^{-1}
Tuapiro 1	2	Q1	11/02/2013	5846597	1860032	Intertidal	22.6 ± 0.2	ND	4.4	9.5	6.4	17	165	0	29
Tuapiro 1	2	Q2	11/02/2013	5846597	1860032	Intertidal	22.6 ± 0.2	ND	4.0	7.8	9.3	22	188	0	26
Tuapiro 1	2	Q3	11/02/2013	5846597	1860032	Intertidal	22.6 ± 0.2	ND	3.9	8.9	8.7	15	134	0	40
Tuapiro 1	2	Q4	11/02/2013	5846597	1860032	Intertidal	22.6 ± 0.2	ND	4.4	9.7	9.7	18	141	0	34
Tuapiro 1	2	Q5	11/02/2013	5846597	1860032	Intertidal	22.6 ± 0.2	ND	3.9	10.1	9.5	13	149	0	20
Tuapiro 1	3	Q1	11/02/2013	5846485	1860748	Intertidal	22.6 ± 0.2	ND	3.0	7.7	10.2	29	351	10	26
Tuapiro 1	3	Q2	11/02/2013	5846485	1860748	Intertidal	22.6 ± 0.2	ND	2.8	6.3	11.2	27	293	10	39
Tuapiro 1	3	Q3	11/02/2013	5846485	1860748	Intertidal	22.6 ± 0.2	ND	2.6	7.6	12.1	23	242	0	66
Tuapiro 1	3	Q4	11/02/2013	5846485	1860748	Intertidal	22.6 ± 0.2	ND	2.7	6.4	11.2	26	343	9	28
Tuapiro 1	3	Q5	11/02/2013	5846485	1860748	Intertidal	22.6 ± 0.2	ND	2.7	12.0	12.4	22	267	17	18
Tuapiro 1	4	Q1	11/02/2013	5847046	1860495	Intertidal	22.6 ± 0.2	ND	3.7	7.0	14.7	15	239	105	359
Tuapiro 1	4	Q2	11/02/2013	5847046	1860495	Intertidal	22.6 ± 0.2	ND	4.3	11.7	15.7	13	294	110	308
Tuapiro 1	4	Q3	11/02/2013	5847046	1860495	Intertidal	22.6 ± 0.2	ND	3.4	9.0	13.8	22	303	134	609
Tuapiro 1	4	Q4	11/02/2013	5847046	1860495	Intertidal	22.6 ± 0.2	ND	3.8	4.9	16.5	15	283	95	501
Tuapiro 1	4	Q5	11/02/2013	5847046	1860495	Intertidal	22.6 ± 0.2	ND	3.6	6.3	16.1	20	599	44	490
Tuapiro 1	5	Q1	11/02/2013	5847350	1860678	Intertidal	22.6 ± 0.2	ND	1.9	4.5	8.8	14	119	12	213
Tuapiro 1	5	Q2	11/02/2013	5847350	1860678	Intertidal	22.6 ± 0.2	ND	1.6	4.3	8.5	16	158	6	169
Tuapiro 1	5	Q3	11/02/2013	5847350	1860678	Intertidal	22.6 ± 0.2	ND	2.2	4.5	10.7	17	176	18	94
Tuapiro 1	5	Q4	11/02/2013	5847350	1860678	Intertidal	22.6 ± 0.2	ND	2.0	3.5	10.0	16	131	20	264
Tuapiro 1	5	Q5	11/02/2013	5847350	1860678	Intertidal	22.6 ± 0.2	ND	2.0	4.1	10.7	17	198	15	60
Tuapiro 2	1	2	27/11/2014	5846678	1860839	Intertidal	20 ± 0.1	31.8	1.5	0.8	9.1	20	91	28	40
Tuapiro 2	1	4	27/11/2014	5846678	1860839	Intertidal	20 ± 0.1	31.8	1.6	0.0	9.0	21	122	33	47
Tuapiro 2	2	6	27/11/2014	5846660	1860848	Intertidal	20 ± 0.1	31.8	1.7	1.2	6.1	16	115	20	43
Tuapiro 2	2	8	27/11/2014	5846660	1860848	Intertidal	20 ± 0.1	31.8	1.8	1.3	6.6	18	131	28	25
Tuapiro 2	3	10	27/11/2014	5846620	1860828	Intertidal	20 ± 0.1	31.8	3.0	3.1	20.3	21	118	35	193
Tuapiro 2	3	12	27/11/2014	5846620	1860828	Intertidal	20 ± 0.1	31.8	3.0	3.9	21.1	14	76	28	224
Tuapiro 2	4	14	27/11/2014	5846603	1860818	Intertidal	20 ± 0.1	31.8	2.8	1.8	14.8	23	135	26	267
Tuapiro 2	4	16	27/11/2014	5846603	1860818	Intertidal	20 ± 0.1	31.8	2.9	1.9	16.1	23	111	31	361
Tuapiro 2	5	18	27/11/2014	5846589	1860812	Intertidal	20 ± 0.1	31.8	3.4	3.7	14.3	17	137	27	406
Tuapiro 2	5	20	27/11/2014	5846589	1860812	Intertidal	20 ± 0.1	31.8	3.4	3.3	13.0	14	79	21	345
Tuapiro 2	6	22	27/11/2014	5846578	1860808	Intertidal	20 ± 0.1	31.8	3.6	7.3	15.6	22	202	38	366
Tuapiro 2	6	24	27/11/2014	5846578	1860808	Intertidal	20 ± 0.1	31.8	3.9	6.8	16.5	14	134	24	424
Tuapiro 2	7	26	27/11/2014	5846609	1860800	Intertidal	20 ± 0.1	31.8	1.5	0.0	6.0	16	53	16	79
Tuapiro 2	7	28	27/11/2014	5846609	1860800	Intertidal	20 ± 0.1	31.8	1.7	0.0	7.4	18	75	21	78
Tuapiro 2	8	30	27/11/2014	5846631	1860811	Intertidal	20 ± 0.1	31.8	1.7	1.2	6.7	21	114	34	114

Study	Site	Plot	Sample date	Location		Water depth	Temperature	Salinity	OC	Mud	Microphytobenthic biomass	Macrofaunal community			DEA
				Latitude	Longitude						Chl*a*	S	N	LB	
units				NZTM	NZTM	m	ºC	ppt	%	%	$\mu g\ g^{-1}$	n $core^{-1}$	n $core^{-1}$	n $core^{-1}$	$\mu mol\ N\ m^{-2}\ h^{-1}$
Tuapiro 2	8	32	27/11/2014	5846631	1860811	Intertidal	20 ± 0.1	31.8	1.8	1.3	11.0	20	124	66	115
Tuapiro 2	9	34	27/11/2014	5846560	1860817	Intertidal	20 ± 0.1	31.8	3.8	10.3	14.2	20	208	22	342
Tuapiro 2	9	36	27/11/2014	5846560	1860817	Intertidal	20 ± 0.1	31.8	4.7	12.0	17.4	17	134	15	387
Tuapiro 2	10	38	27/11/2014	5846549	1860828	Intertidal	20 ± 0.1	31.8	5.1	14.5	18.1	18	142	26	528
Tuapiro 2	10	40	27/11/2014	5846549	1860828	Intertidal	20 ± 0.1	31.8	4.6	12.3	15.3	16	154	21	435
Tuapiro 2	11	42	27/11/2014	5846538	1860828	Intertidal	20 ± 0.1	31.8	5.4	21.6	20.0	19	100	14	601
Tuapiro 2	11	44	27/11/2014	5846538	1860828	Intertidal	20 ± 0.1	31.8	5.5	20.2	19.8	15	123	15	519
Tuapiro 2	12	46	27/11/2014	5846536	1860850	Intertidal	20 ± 0.1	31.8	3.9	15.2	12.9	21	149	27	411
Tuapiro 2	12	48	27/11/2014	5846536	1860850	Intertidal	20 ± 0.1	31.8	4.1	12.7	13.3	18	164	19	467
Kaipara	1		17/03/2014	5971943	1715904	Intertidal	20.5 ± 0.04	33.9*	1.9	10.0	13.2	29	225	25	51
Kaipara	2		17/03/2014	5971771	1715908	Intertidal	20.5 ± 0.04	33.9*	0.9	0.0	12.7	19	92	43	4
Kaipara	3		17/03/2014	5971577	1715908	Intertidal	20.5 ± 0.04	33.9*	1.7	7.3	14.6	26	332	116	135
Kaipara	4		17/03/2014	5971494	1715904	Intertidal	20.5 ± 0.04	33.9*	1.5	1.1	12.5	19	107	1	189
Kaipara	5		17/03/2014	5971296	1715921	Intertidal	20.5 ± 0.04	33.9*	1.2	9.9	6.0	29	174	5	117
Kaipara	6		17/03/2014	5971196	1715921	Intertidal	20.5 ± 0.04	33.9*	1.5	4.9	7.9	22	137	22	199
Kaipara	7		17/03/2014	5971171	1715922	Intertidal	20.5 ± 0.04	33.9*	0.6	0.0	6.1	13	47	2	4
Kaipara	8		17/03/2014	5971091	1715923	Intertidal	20.5 ± 0.04	33.9*	0.8	0.0	5.5	19	72	15	6
Kaipara	9		17/03/2014	5971015	1715922	Intertidal	20.5 ± 0.04	33.9*	1.0	13.6	7.0	26	130	22	14
Kaipara	10		17/03/2014	5970999	1716025	Intertidal	20.5 ± 0.04	33.9*	1.1	8.7	23.2	28	340	6	39
Kaipara	11		17/03/2014	5971315	1716004	Intertidal	20.5 ± 0.04	33.9*	1.0	4.1	3.6	24	419	16	28
Kaipara	12		17/03/2014	5971518	1715991	Intertidal	20.5 ± 0.04	33.9*	1.2	4.1	7.8	18	126	20	38
Kaipara	13		17/03/2014	5971672	1715985	Intertidal	20.5 ± 0.04	33.9*	0.8	0.0	9.8	19	111	35	10
Kaipara	14		17/03/2014	5971755	1715978	Intertidal	20.5 ± 0.04	33.9*	0.8	0.0	12.1	14	53	16	6
Kaipara	15		17/03/2014	5971813	1715977	Intertidal	20.5 ± 0.04	33.9*	0.8	0.0	11.7	14	90	34	6
Kaipara	16		17/03/2014	5971935	1716072	Intertidal	20.5 ± 0.04	33.9*	0.7	0.0	5.3	10	79	29	7
Kaipara	17		17/03/2014	5971835	1716072	Intertidal	20.5 ± 0.04	33.9*	0.8	3.5	8.6	17	127	10	3
Kaipara	18		17/03/2014	5971656	1716075	Intertidal	20.5 ± 0.04	33.9*	0.8	0.0	10.3	17	87	41	6
Kaipara	19		17/03/2014	5971559	1716079	Intertidal	20.5 ± 0.04	33.9*	0.7	0.0	5.9	14	54	32	10
Kaipara	20		17/03/2014	5971224	1716111	Intertidal	20.5 ± 0.04	33.9*	1.7	14.5	14.5	25	108	5	225
Kaipara	21		17/03/2014	5971055	1716120	Intertidal	20.5 ± 0.04	33.9*	1.2	2.5	7.2	28	142	17	138
Kaipara	22		17/03/2014	5971094	1716225	Intertidal	20.5 ± 0.04	33.9*	1.6	9.2	13.7	24	136	6	209
Kaipara	23		17/03/2014	5971177	1716216	Intertidal	20.5 ± 0.04	33.9*	2.0	12.2	12.8	27	203	7	373
Kaipara	24		17/03/2014	5971253	1716205	Intertidal	20.5 ± 0.04	33.9*	1.4	4.5	20.9	25	88	6	73
Kaipara	25		17/03/2014	5971446	1716172	Intertidal	20.5 ± 0.04	33.9*	0.7	0.0	5.7	17	95	39	10
Kaipara	26		17/03/2014	5971463	1716167	Intertidal	20.5 ± 0.04	33.9*	0.6	0.0	4.2	12	38	16	5

Study	Site	Plot	Sample date	Location		Water depth	Temperature	Salinity	OC	Mud	Microphytobenthic biomass	Macrofaunal community			DEA
				Latitude	Longitude						Chl*a*	S	N	LB	
units				NZTM	NZTM	m	ºC	ppt	%	%	µg g^{-1}	n core^{-1}	n core^{-1}	n core^{-1}	µmol N m^{-2} h^{-1}
Kaipara	27		17/03/2014	5971774	1716143	Intertidal	20.5 ± 0.04	33.9*	0.7	0.0	9.6	11	50	13	4
Kaipara	28		17/03/2014	5971955	1716127	Intertidal	20.5 ± 0.04	33.9*	0.7	0.0	8.9	9	19	4	20
Mahurangi	1	A	5/03/2015	5958958	1755223	4.9	23.1 ± 0.1	35.0	5.7	37.2	6.6	8	50	0	877
Mahurangi	1	B	5/03/2015	5958958	1755223	4.9	23.1 ± 0.1	35.0	5.2	35.0	7.1	12	58	0	995
Mahurangi	1	C	5/03/2015	5958958	1755223	4.9	23.1 ± 0.1	35.0	5.4	40.6	2.6	4	53	0	844
Mahurangi	1	D	5/03/2015	5958958	1755223	4.9	23.1 ± 0.1	35.0	5.7	37.5	6.8	7	82	0	957
Mahurangi	2	A	5/03/2015	5960410	1754366	5.3	23.1 ± 0.1	35.0	4.4	52.2	8.3	19	184	0	594
Mahurangi	2	B	5/03/2015	5960410	1754366	5.3	23.1 ± 0.1	35.0	4.9	42.7	7.8	14	226	0	604
Mahurangi	2	C	5/03/2015	5960410	1754366	5.3	23.1 ± 0.1	35.0	4.5	38.0	6.0	15	195	0	701
Mahurangi	2	D	5/03/2015	5960410	1754366	5.3	23.1 ± 0.1	35.0	4.8	42.5	4.3	24	263	0	650
Mahurangi	3	A	5/03/2015	5962275	1754645	4.5	23.1 ± 0.1	35.0	3.7	19.5	6.7	11	164	0	972
Mahurangi	3	B	5/03/2015	5962275	1754645	4.5	23.1 ± 0.1	35.0	3.8	25.7	7.4	16	147	2	698
Mahurangi	3	C	5/03/2015	5962275	1754645	4.5	23.1 ± 0.1	35.0	3.8	27.2	7.5	19	186	0	798
Mahurangi	3	D	5/03/2015	5962275	1754645	4.5	23.1 ± 0.1	35.0	3.8	25.9	6.5	17	184	0	1027
Mahurangi	4	A	5/03/2015	5964378	1753855	3.8	23.1 ± 0.1	35.0	4.1	20.6	9.9	17	148	0	752
Mahurangi	4	B	5/03/2015	5964378	1753855	3.8	23.1 ± 0.1	35.0	4.2	24.9	9.2	18	151	0	716
Mahurangi	4	C	5/03/2015	5964378	1753855	3.8	23.1 ± 0.1	35.0	4.5	24.7	9.8	11	138	0	832
Mahurangi	4	D	5/03/2015	5964378	1753855	3.8	23.1 ± 0.1	35.0	3.9	16.7	8.2	17	146	0	963
Whitford	A	1	1/03/2018	5913608	1774416	Intertidal	23.4 ± 0.1	33.9 ± 0.04	0.8	3.8	6.6	14	273	34	70
Whitford	A	2	1/03/2018	5913591	1774458	Intertidal	23.4 ± 0.1	33.9 ± 0.04	0.5	0.6	6.5	12	77	19	70
Whitford	A	3	1/03/2018	5913570	1774500	Intertidal	23.4 ± 0.1	33.9 ± 0.04	0.5	0.4	6.4	6	8	3	25
Whitford	A	4	1/03/2018	5913830	1776291	Intertidal	23.4 ± 0.1	33.9 ± 0.04	1.1	8.9	8.2	6	21	12	8
Whitford	A	5	1/03/2018	5913823	1776217	Intertidal	23.4 ± 0.1	33.9 ± 0.04	0.4	0.8	4.3	6	8	4	3
Whitford	A	6	1/03/2018	5913818	1776146	Intertidal	23.4 ± 0.1	33.9 ± 0.04	0.4	0.5	3.9	3	10	8	4
Whitford	A	7	1/03/2018	5913193	1773828	Intertidal	23.4 ± 0.1	33.9 ± 0.04	1.9	21.3	16.9	9	24	8	2
Whitford	A	8	1/03/2018	5913235	1773880	Intertidal	23.4 ± 0.1	33.9 ± 0.04	1.9	14.5	12.8	16	307	66	655
Whitford	A	9	1/03/2018	5913274	1774090	Intertidal	23.4 ± 0.1	33.9 ± 0.04	2.1	22.7	11.3	9	27	8	268
Whitford	A	10	1/03/2018	5913278	1774159	Intertidal	23.4 ± 0.1	33.9 ± 0.04	2.1	13.6	17.3	14	194	92	249
Whitford	A	11	1/03/2018	5913246	1774434	Intertidal	23.4 ± 0.1	33.9 ± 0.04	1.0	7.5	14.0	14	288	40	380
Whitford	A	12	1/03/2018	5913279	1774480	Intertidal	23.4 ± 0.1	33.9 ± 0.04	0.5	2.1	7.7	9	50	21	306
Whitford	A	13	1/03/2018	5913312	1774520	Intertidal	23.4 ± 0.1	33.9 ± 0.04	0.5	0.8	4.9	9	17	2	27
Whitford	A	14	1/03/2018	5913603	1775091	Intertidal	23.4 ± 0.1	33.9 ± 0.04	0.8	0.6	5.7	7	26	1	13
Whitford	A	15	1/03/2018	5913589	1775191	Intertidal	23.4 ± 0.1	33.9 ± 0.04	0.4	0.3	5.7	11	40	13	14
Whitford	A	16	1/03/2018	5913647	1775200	Intertidal	23.4 ± 0.1	33.9 ± 0.04	0.4	0.5	7.1	9	23	5	17
Whitford	A	17	1/03/2018	5913668	1775112	Intertidal	23.4 ± 0.1	33.9 ± 0.04	0.5	0.5	6.4	10	80	19	8

Study	Site	Plot	Sample date	Location		Water depth	Temperature	Salinity	OC	Mud	Microphytobenthic biomass	Macrofaunal community			DEA
				Latitude	Longitude						Chl*a*	S	N	LB	
units				NZTM	NZTM	m	ºC	ppt	%	%	µg g^{-1}	n core^{-1}	n core^{-1}	n core^{-1}	µmol N m^{-2} h^{-1}
Whitford	A	18	1/03/2018	5913697	1775702	Intertidal	23.4 ± 0.1	33.9 ± 0.04	0.6	0.6	5.2	2	4	3	6
Whitford	A	19	1/03/2018	5913622	1775629	Intertidal	23.4 ± 0.1	33.9 ± 0.04	0.4	0.6	6.2	5	24	17	9
Whitford	A	20	1/03/2018	5913621	1775686	Intertidal	23.4 ± 0.1	33.9 ± 0.04	0.5	0.9	6.2	10	72	20	1
Whitford	B	1	1/03/2018	5913608	1774416	Intertidal	21 ± 0.1	33.5 ± 0.24	1.0	2.6	7.2	18	329	31	65
Whitford	B	2	1/03/2018	5913591	1774458	Intertidal	21 ± 0.1	33.5 ± 0.24	0.6	0.5	6.4	8	32	10	14
Whitford	B	3	1/03/2018	5913570	1774500	Intertidal	21 ± 0.1	33.5 ± 0.24	0.4	0.4	6.3	7	20	6	9
Whitford	B	4	1/03/2018	5913830	1776291	Intertidal	21 ± 0.1	33.5 ± 0.24	0.9	6.2	7.7	11	37	13	67
Whitford	B	5	1/03/2018	5913823	1776217	Intertidal	21 ± 0.1	33.5 ± 0.24	0.4	0.6	4.5	4	10	4	3
Whitford	B	6	1/03/2018	5913818	1776146	Intertidal	21 ± 0.1	33.5 ± 0.24	0.5	0.4	4.6	4	5	1	3
Whitford	B	7	1/03/2018	5912230	1774984	Intertidal	21 ± 0.1	33.5 ± 0.24	4.0	36.2	18.2	5	20	0	783
Whitford	B	8	1/03/2018	5912348	1774871	Intertidal	21 ± 0.1	33.5 ± 0.24	2.6	24.1	15.5	8	30	1	788
Whitford	B	9	1/03/2018	5912945	1774793	Intertidal	21 ± 0.1	33.5 ± 0.24	0.5	4.2	4.4	3	6	4	9
Whitford	B	10	1/03/2018	5913029	1774802	Intertidal	21 ± 0.1	33.5 ± 0.24	0.4	0.8	5.7	5	21	6	7
Whitford	B	11	1/03/2018	5913188	1774786	Intertidal	21 ± 0.1	33.5 ± 0.24	0.5	5.3	4.8	5	8	4	72
Whitford	B	12	1/03/2018	5913249	1774783	Intertidal	21 ± 0.1	33.5 ± 0.24	1.0	0.9	6.2	7	31	6	9
Whitford	B	13	1/03/2018	5913304	1774779	Intertidal	21 ± 0.1	33.5 ± 0.24	0.4	0.2	6.9	6	9	2	7
Whitford	B	14	1/03/2018	5913396	1775253	Intertidal	21 ± 0.1	33.5 ± 0.24	4.6	53.7	17.8	5	11	0	534
Whitford	B	15	1/03/2018	5913425	1775210	Intertidal	21 ± 0.1	33.5 ± 0.24	4.8	62.6	20.6	6	186	0	592
Whitford	B	18	1/03/2018	5913604	1774750	Intertidal	21 ± 0.1	33.5 ± 0.24	0.4	0.2	5.4	12	38	4	16
Whitford	B	19	1/03/2018	5913676	1774775	Intertidal	21 ± 0.1	33.5 ± 0.24	0.4	0.3	6.9	10	43	3	15
Whitford	B	20	1/03/2018	5913653	1774867	Intertidal	21 ± 0.1	33.5 ± 0.24	0.4	0.5	7.4	9	26	6	18
Whitford	C	1	1/03/2018	5913608	1774416	Intertidal	24.1 ± 0.1	33.7 ± 0.1	0.5	2.5	7.7	19	249	30	50
Whitford	C	2	1/03/2018	5913591	1774458	Intertidal	24.1 ± 0.1	33.7 ± 0.1	0.4	0.6	6.6	11	43	13	17
Whitford	C	3	1/03/2018	5913570	1774500	Intertidal	24.1 ± 0.1	33.7 ± 0.1	0.4	0.5	6.9	7	13	4	20
Whitford	C	4	1/03/2018	5913830	1776291	Intertidal	24.1 ± 0.1	33.7 ± 0.1	0.7	7.5	7.6	14	55	26	64
Whitford	C	5	1/03/2018	5913823	1776217	Intertidal	24.1 ± 0.1	33.7 ± 0.1	0.5	0.8	5.5	5	12	7	3
Whitford	C	6	1/03/2018	5913818	1776146	Intertidal	24.1 ± 0.1	33.7 ± 0.1	0.3	0.5	4.4	3	3	1	3
Whitford	C	7	1/03/2018	5913811	1775350	Intertidal	24.1 ± 0.1	33.7 ± 0.1	0.4	0.4	5.6	8	15	5	3
Whitford	C	8	1/03/2018	5913822	1775425	Intertidal	24.1 ± 0.1	33.7 ± 0.1	0.4	0.5	4.4	7	21	12	3
Whitford	C	9	1/03/2018	5913461	1776945	Intertidal	24.1 ± 0.1	33.7 ± 0.1	0.5	2.8	4.4	7	14	4	0
Whitford	C	10	1/03/2018	5913504	1776932	Intertidal	24.1 ± 0.1	33.7 ± 0.1	0.6	1.4	4.6	5	8	2	3
Whitford	C	11	1/03/2018	5913520	1776243	Intertidal	24.1 ± 0.1	33.7 ± 0.1	0.6	1.0	6.0	7	30	0	9
Whitford	C	12	1/03/2018	5913577	1776234	Intertidal	24.1 ± 0.1	33.7 ± 0.1	0.5	2.4	4.5	12	59	13	8
Whitford	C	13	1/03/2018	5913628	1776227	Intertidal	24.1 ± 0.1	33.7 ± 0.1	0.6	4.9	4.1	10	37	15	35
Whitford	C	14	1/03/2018	5913500	1776594	Intertidal	24.1 ± 0.1	33.7 ± 0.1	0.5	1.3	4.6	5	7	4	6

Study	Site	Plot	Sample date	Location		Water depth	Temperature	Salinity	OC	Mud	Microphytobenthic biomass	Macrofaunal community			DEA
				Latitude	Longitude						Chl*a*	S	N	LB	
units				NZTM	NZTM	m	ºC	ppt	%	%	µg g^{-1}	n $core^{-1}$	n $core^{-1}$	n $core^{-1}$	µmol N m^{-2} h^{-1}
Whitford	C	15	1/03/2018	5913501	1776643	Intertidal	24.1 ± 0.1	33.7 ± 0.1	0.5	1.2	6.2	7	21	7	5
Whitford	C	16	1/03/2018	5913467	1776618	Intertidal	24.1 ± 0.1	33.7 ± 0.1	0.4	1.6	3.9	8	14	5	5
Whitford	C	17	1/03/2018	5913496	1776985	Intertidal	24.1 ± 0.1	33.7 ± 0.1	0.5	1.5	3.8	6	8	2	3
Whitford	C	18	1/03/2018	5913491	1777193	Intertidal	24.1 ± 0.1	33.7 ± 0.1	0.9	12.2	111	12	30	6	205
Whitford	C	19	1/03/2018	5913500	1777233	Intertidal	24.1 ± 0.1	33.7 ± 0.1	2.2	43.5	9.5	8	21	0	453
Whitford	C	20	1/03/2018	5913523	1777267	Intertidal	24.1 ± 0.1	33.7 ± 0.1	1.1	18.0	7.1	14	273	1	394

Abbreviations: OC: sediment organic content, Mud: sediment mud content, Chl*a*: chlorophyll *a* content, S: number of taxa, N: total abundance, LB: number of large bivalves *A. stutchburyi* and *M. liliana*, DEA: denitrification enzyme activity, ND: no data. *Salinity data were site averages during high tide on the day of sampling or other best estimate value given available data, for Kaipara, salinity value was from another study at the same location. Study: Waikareao (unpublished survey data), Tuapiro 1 (unpublished survey data), Tuapiro 2 (ambient data from Douglas, et al. [1]), Kaipara (ambient data from Douglas, et al. [2]), Mahurangi (survey data from O'Meara, et al. [3]), Whitford (survey data from Lohrer, et al. [4]).

Table S2. Expected potential relationships between structural equation model variables based on literature. Path numbers correspond to those in Figure 4. Asterisks (*) indicate empirical work carried out in New Zealand estuaries. Abbreviations: Microphytobenthos (MPB).

Path number	Variable 1	Variable 2	Relationship	Source
1	Mud content	Organic content	Co-occurrence of fine particles	Pratt, et al. [5]*, Huettel and Rusch [6], Sloth, et al. [7]
2	Mud content	DEA	DEA increases with increased mud content	Douglas, et al. [1]*
3	Organic content	DEA	Organic matter provides a carbon source for denitrification therefore positively influences DEA	Caffrey, et al. [8], Eyre, et al. [9], Knowles [10], Cornwell, et al. [11]
4	Chlorophyll *a*	DEA	Photosynthetic activity by MPB can enhance coupled nitrification-denitrification by oxygenating sediments. MPB may decrease denitrification through competition for nutrients.	Marzocchi, et al. [12], Decleyre, et al. [13], Risgaard-Petersen, et al. [14], Sundback, et al. [15]
5	Macrofunal abundance	DEA	Density of macrofauna influences denitrification	Douglas, et al. [2]*, Braeckman, et al. [16]
6	Macrofaunal diversity	DEA	Diversity of macrofauna can enhance denitrification	Douglas, et al. [2]*, Braeckman, et al. [17]
7	Large bivalves	DEA	Large bioturbating fauna enhance denitrification through their influence on sediment porewater transfer and oxygen dynamics. They can also increase sediment organic matter via biodeposition.	Douglas, et al. [2]*, Woodin, et al. [18]*, Aller [19], Pelegri, et al. [20], Braeckman, et al. [16], Jones, et al. [21]*
8	Mud	Chlorophyll *a*	Muddy sediments tend to have higher MPB biomass, but high levels of fine sediments can have a negative effect on MPB biomass.	Pratt, et al. [22]*, Cahoon, et al. [23]*, van de Koppel, et al. [24]
9	Organic content	Chlorophyll *a*	Sediment organic content and Chlorophyll a are positively correlated. Degradation of MPB contribute to sediment organic matter pool	Ehrenhauss, et al. [25], Pratt, et al. [5]*, Miller, et al. [26]
10	Macrofaunal abundance	Chlorophyll *a*	MPB provide a food source for many benthic macrofauna and are usually positively correlated.	Pratt, et al. [5]*, Miller, et al. [26], Rodil, et al. [27]*
11	Macrofaunal diversity	Chlorophyll *a*	MPB provide a food source for many benthic macrofauna and are usually positively correlated.	Pratt, et al. [5]*, Miller, et al. [26]
12	Large bivalves	Chlorophyll *a*	MPB provide a food source for large bivalves and have a positive effect on abundance of *M. liliana* and *A. stutchburyi*. *A. stutchburyi* strongly influence MPB productivity.	Pratt, et al. [28]*, Pratt, et al. [22]*, Thrush, et al. [29]*, Lelieveld, et al. [30]*, Van Colen, et al. [31]*, Sandwell, et al. [32]*, Van Colen, et al. [33], Harris, et al. [34]*,

13	Mud	Macrofaunal diversity	Fine sediments have a negative impact on macrofaunal diversity, usually above a threshold. Many species show an ‘optimum’ level of mud content.	Pratt, et al. [5]*, Anderson [35]*, Cummings, et al. [36]*
14	Organic content	Macrofaunal diversity	Sediment organic content provides a food source for macrofauna so can be positively correlated with diversity. However, an overabundance of organic matter can negatively impact macrofaunal diversity.	Karlson, et al. [37], Welsh [38], Hyland, et al. [39]
15	Macrofaunal abundance	Macrofaunal diversity	Positively correlated (covariance)	Pratt, et al. [5]*, Robertson, et al. [40]*, Anderson [35]*, Thrush, et al. [41]*
16	Mud	Macrofaunal abundance	Fine sediments have a negative impact on macrofaunal abundance, usually above a threshold. Many species show an ‘optimum’ level of mud content.	Pratt, et al. [5]*, Anderson [35]*, Cummings, et al. [36]*
17	Organic content	Macrofaunal abundance	Macrofauna contribute to organic detritus in the sediments through feeding and breakdown of particulate matter and biodeposition. However, an overabundance of organic matter can negatively impact macrofaunal abundance.	Rhoads and Boyer [42], Welsh [38], Hyland, et al. [39]
18	Large bivalves	Macrofaunal abundance	*M. liliana* and *A. stutchburyi* density can influence the density of other species (both negative and positive).	Van Colen, et al. [31]*, Thrush, et al. [43]*, Thrush, et al. [29]*, Harris, et al. [34]*
19	Mud	Large bivalves	Fine sediments negatively impact abundance of *M. liliana* and *A. stutchburyi*.	Pratt, et al. [5]*, Thrush, et al. [41]*, Thrush, et al. [44]*, Cummings, et al. [45]*
20	Organic content	Large bivalves	Sediment organic content provides a food source for *M. liliana.*	Rhoads and Boyer [42], Thrush, et al. [29]*
21	Large bivalves	Macrofaunal diversity	*M. liliana* and *A. stutchburyi* density can influence the density of other species (both negative and positive).	Thrush, et al. [43]*, Van Colen, et al. [33]

Table S2 REFERENCES

1 Douglas, E. J. *et al.* Sedimentary environment influences ecosystem response to nutrient enrichment. *Estuaries and Coasts* **41**, 1994-2008, doi:10.1007/s12237-018-0416-5 (2018).

2 Douglas, E. J. *et al.* Macrofaunal functional diversity provides resilience to nutrient enrichment in coastal sediments. *Ecosystems* **20**, 1324-1336, doi:10.1007/s10021-017-0113-4 (2017).

3 O'Meara, T., Thrush, S., Hewitt, J., Douglas, E. J. & Lohrer, A. Denitrification and the role of macrofauna along a head-to-mouth estuarine gradient. *Estuaries and Coasts* (In review).

4 Lohrer, A., Stephenson, F., Douglas, E. J. & Townsend, M. Mapping the estuarine ecosystem service of pollutant removal using empirically validated boosted regression tree models. *Ecological Applications* (In review).

5 Pratt, D. R., Lohrer, A. M., Pilditch, C. A. & Thrush, S. F. Changes in ecosystem function across sedimentary gradients in estuaries. *Ecosystems* **17**, 182-194, doi:10.1007/s10021-013-9716-6 (2013).

6 Huettel, M. & Rusch, A. Transport and degradation of phytoplankton in permeable sediment. *Limnology and Oceanography* **45**, 534-549, doi:10.4319/lo.2000.45.3.0534 (2000).

7 Sloth, N. P., Blackburn, H., Hansen, L. S., Risgaard-Pedersen, N. & Lomstein, B. A. Nitrogen cycling in sediments with different organic loading. *Marine Ecology Progress Series* **116**, 163-170 (1995).

8 Caffrey, J. M., Sloth, N. P., Kaspar, H. F. & Blackburn, T. H. Effect of organic loading on nitrification and denitrification in a marine sediment microcosm. *FEMS Microbiology Ecology* **12**, 159-167, doi:10.1016/0168-6496(93)90011-u (1993).

9 Eyre, B. D., Maher, D. T. & Squire, P. Quantity and quality of organic matter (detritus) drives N_2 effluxes (net denitrification) across seasons, benthic habitats and estuaries. *Global Biogeochemical Cycles* **27**, 1083–1095, doi:10.1002/2013GB004631 (2013).

10 Knowles, R. Denitrification. *Microbiological Reviews* **46**, 43-70 (1982).

11 Cornwell, J. C., Kemp, W. M. & Kana, T. M. Denitrification in coastal ecosystems: methods, environmental controls, and ecosystem level controls, a review. *Aquatic Ecology* **33**, 41-54, doi:10.1023/A:1009921414151 (1999).

12 Marzocchi, U., Thamdrup, B., Stief, P. & Glud, R. N. Effect of settled diatom-aggregates on benthic nitrogen cycling. *Limnology and Oceanography* **63**, 431-444, doi:10.1002/lno.10641 (2018).

13 Decleyre, H. *et al.* A doubling of microphytobenthos biomass coincides with a tenfold increase in denitrifier and total bacterial abundances in intertidal sediments of a temperate estuary. *PLoS ONE* **10**, e0126583, doi:10.1371/journal.pone.0126583 (2015).

14 Risgaard-Petersen, N., Rysgaard, S., Nielsen, L. P. & Revsbech, N. P. Diurnal-variation of denitrification and nitrification in sediments colonized by benthic microphytes. *Limnology and Oceanography* **39**, 573-579 (1994).

15 Sundback, K., Miles, A. & Goransson, E. Nitrogen fluxes, denitrification and the role of microphytobenthos in microtidal shallow-water sediments: An annual study. *Marine Ecology Progress Series* **200**, 59-76, doi:10.3354/meps200059 (2000).

16 Braeckman, U. *et al.* Role of macrofauna functional traits and density in biogeochemical fluxes and bioturbation. *Marine Ecology Progress Series* **399**, 173-186, doi:10.3354/meps08336 (2010).

17 Braeckman, U. *et al.* Variable importance of macrofaunal functional biodiversity for biogeochemical cycling in temperate coastal sediments. *Ecosystems* **17**, 720-737, doi:10.1007/s10021-014-9755-7 (2014).

18 Woodin, S. A. *et al.* Same pattern, different mechanism: Locking onto the role of key species in seafloor ecosystem process. *Scientific Reports* **6**, 26678, doi:10.1038/srep26678 (2016).

19 Aller, R. C. in *Nitrogen Cycling in Coastal Marine Environments* (eds T. H. Blackburn & J. Sorensen) Ch. 13, 301-338 (John Wiley & Sons, 1988).

20 Pelegri, S. P., Nielsen, L. P. & Blackburn, T. H. Denitrification in estuarine sediment stimulated by the irrigation activity of the amphipod *Corophium volutator*. *Marine Ecology Progress Series* **105**, 285-290, doi:10.3354/meps105285 (1994).

21 Jones, H. F. E., Pilditch, C. A., Bruesewitz, D. A. & Lohrer, A. M. Sedimentary environment influences the effect of an infaunal suspension feeding bivalve on estuarine ecosystem function. *PLoS One* **6**, e27065, doi:10.1371/journal.pone.0027065 (2011).

22 Pratt, D. R., Pilditch, C. A., Lohrer, D. & Thrush, S. F. The effects of short-term increases in turbidity on sandflat microphytobenthic productivity and nutrient fluxes. *Journal of Sea Research* **92**, 170-177, doi:10.1016/j.seares.2013.07.009 (2014).

23 Cahoon, L. B., Nearhoof, J. E. & Tilton, C. L. Sediment grain size effect on benthic microalgal biomass in shallow aquatic ecosystems. *Estuaries* **22**, 735-741, doi:10.2307/1353106 (1999).

24 van de Koppel, J., Herman, P. M. J., Thoolen, P. & Heip, C. H. R. Do alternate stable states occur in natural ecosystems? Evidence from a tidal flat. *Ecology* **82**, 3449-3461, doi:doi:10.1890/0012-9658(2001)082[3449:DASSOI]2.0.CO;2 (2001).
25 Ehrenhauss, S., Witte, U., Janssen, F. & Huettel, M. Decomposition of diatoms and nutrient dynamics in permeable North Sea sediments. *Continental Shelf Research* **24**, 721-737, doi:10.1016/j.csr.2004.01.002 (2004).
26 Miller, D. C., Geider, R. J. & MacIntyre, H. L. Microphytobenthos: The ecological role of the "secret garden" of unvegetated, shallow-water marine habitats. II. role in sediment stability and shallow-water food webs. *Estuaries* **19**, 202-212, doi:10.2307/1352225 (1996).
27 Rodil, I. F., Lohrer, A. M. & Thrush, S. F. Sensitivity of heterogeneous marine benthic habitats to subtle stressors. *PLOS ONE* **8**, e81646, doi:10.1371/journal.pone.0081646 (2013).
28 Pratt, D. R. *et al.* Detecting subtle shifts in ecosystem functioning in a dynamic estuarine environment. *PLoS One* **10**, e0133914, doi:10.1371/journal.pone.0133914 (2015).
29 Thrush, S. F., Hewitt, J. E., Gibbs, M., Lundquist, C. & Norkko, A. Functional role of large organisms in intertidal communities: Community effects and ecosystem function. *Ecosystems* **9**, 1029-1040, doi:10.1007/s10021-005-0068-8 (2006).
30 Lelieveld, S. D., Pilditch, C. A. & Green, M. O. Effects of deposit-feeding bivalve (*Macomona liliana*) density on intertidal sediment stability. *New Zealand Journal of Marine and Freshwater Research* **38**, 115-128 (2004).
31 Van Colen, C. *et al.* Bottom–up and top–down mechanisms indirectly mediate interactions between benthic biotic ecosystem components. *Journal of Sea Research* **98**, 42-48, doi:10.1016/j.seares.2014.10.016 (2014).
32 Sandwell, D. R., Pilditch, C. A. & Lohrer, A. M. Density dependent effects of an infaunal suspension-feeding bivalve (*Austrovenus stutchburyi*) on sandflat nutrient fluxes and microphytobenthic productivity. *Journal of Experimental Marine Biology and Ecology* **373**, 16-25, doi:10.1016/j.jembe.2009.02.015 (2009).
33 Van Colen, C., Thrush, S. F., Vincx, M. & Ysebaert, T. Conditional responses of benthic communities to interference from an intertidal bivalve. *PLOS ONE* **8**, e65861, doi:10.1371/journal.pone.0065861 (2013).
34 Harris, R. *et al.* Biotic interactions influence sediment erodibility on wave-exposed sandflats. *Marine Ecology Progress Series* **523**, 15-30, doi:10.3354/meps11164 (2015).
35 Anderson, M. J. Animal-sediment relationships revisited: Characterising species' distributions along an environmental gradient using canonical analysis and quantile regression splines. *Journal of Experimental Marine Biology and Ecology* **366**, 16-27 (2008).
36 Cummings, V., Thrush, S., Hewitt, J., Norkko, A. & Pickmere, S. Terrestrial deposits on intertidal sandflats: sediment characteristics as indicators of habitat suitability for recolonising macrofauna. *Marine Ecology Progress Series* **253**, 39-54, doi:10.3354/meps253039 (2003).
37 Karlson, K., Bonsdorff, E. & Rosenberg, R. The impact of benthic macrofauna for nutrient fluxes from Baltic Sea sediments. *Ambio* **36**, 161-167, doi:10.1579/0044-7447(2007)36[161:tiobmf]2.0.co;2 (2007).
38 Welsh, D. T. It's a dirty job but someone has to do it: The role of marine benthic macrofauna in organic matter turnover and nutrient recycling to the water column. *Chemistry and Ecology* **19**, 321-342, doi:10.1080/0275754031000155474 (2003).
39 Hyland, J. *et al.* Organic carbon of sediments as an indicator of stress in the marine benthos. *Marine Ecology Progress Series* **295**, 91-103, doi:10.3354/meps295091 (2005).
40 Robertson, B. P., Gardner, J. P. A. & Savage, C. Macrobenthic–mud relations strengthen the foundation for benthic index development: A case study from shallow, temperate New Zealand estuaries. *Ecological Indicators* **58**, 161-174, doi:10.1016/j.ecolind.2015.05.039 (2015).
41 Thrush, S. F. *et al.* Habitat change in estuaries: Predicting broad-scale responses of intertidal macrofauna to sediment mud content. *Marine Ecology Progress Series* **263**, 101-112, doi:10.3354/meps263101 (2003).
42 Rhoads, D. & Boyer, L. in *Animal-sediment relations* Vol. 100 (eds PL McCall & MJS Tevesz) 3-52 (Springer, 1982).
43 Thrush, S. F. *et al.* Matching the outcome of small-scale density manipulation experiments with larger scale patterns: an example of bivalve adult/juvenile interactions. *Journal of Experimental Marine Biology and Ecology* **216**, 153-169, doi:10.1016/S0022-0981(97)00094-4 (1997).
44 Thrush, S. F. *et al.* Muddy waters: Elevating sediment input to coastal and estuarine habitats. *Frontiers in Ecology and the Environment* **2**, 299-306, doi:10.2307/3868405 (2004).
45 Cummings, V., Vopel, K. & Thrush, S. Terrigenous deposits in coastal marine habitats: influences on sediment geochemistry and behaviour of post-settlement bivalves. *Marine Ecology Progress Series* **383**, 173-185, doi:10.3354/meps07983 (2009).
